# Time to Address Eating Disorder Risk Among Medical Students

**DOI:** 10.3390/bs16060899

**Published:** 2026-06-02

**Authors:** Sarah Mabee, Harrison Blefeld, Wyatt Mayer, Paul Zarutskie, Owen Kelly

**Affiliations:** College of Osteopathic Medicine, Sam Houston State University, Conroe, TX 77304, USA; sjm097@shsu.edu (S.M.); hxb043@shsu.edu (H.B.); wcm011@shsu.edu (W.M.); pwz001@shsu.edu (P.Z.)

**Keywords:** eating disorder, medical students, mental health, chronic stress, body image, physical activity, curriculum

## Abstract

This narrative review examines the prevalence and potential key causes of eating disorder risk among medical students across countries and training environments. Medical students frequently demonstrate a higher prevalence of eating disorder risk compared to the general population. Rates vary by instrument, geography, stage of training, and sample composition. Recurrent correlates include female gender, body image dissatisfaction, perfectionism, stress, anxiety, depression, lack of physical activity and problematic social media use. The review also highlights important methodological limitations in the literature, including predominantly cross-sectional design, heterogeneous screening tools, inconsistent reporting of all variables, limited longitudinal follow-up, and sparse inclusion of gender-diverse populations. Overall, the evidence supports eating disorder risk as an important medical student wellness issue while also showing that no single factor fully explains the observed burden. The findings support systemic changes in universities to help prevent eating disorders, including better student health education, confidential services and prioritizing student wellness. Longitudinal research is urgently needed to clarify modifiable risk factors as they relate to chronic stress in medical school and inform targeted prevention strategies.

## 1. Introduction

Eating disorders are silent and enigmatic, and present as complex psychological issues (Iveland, 2024; Thomas & Becker, 2021). Eating disorder is the umbrella term used to describe a host of behavioral conditions, including anorexia nervosa (AN), bulimia nervosa (BN), binge eating disorder (BED), and other specified feeding or eating disorders (OSFEDs) (American Psychiatric Association, 2000). Clinically, they are characterized by persistent disturbances in eating behavior, including restriction, binge eating, or compensatory behaviors such as purging, that are typically accompanied by an excessive preoccupation with body weight or shape. Key characteristics include body dissatisfaction, anxiety about eating, a drive for thinness, fear of losing control or weight gain, an immense feeling of guilt/shame, and a need for perfectionism (Ortiz et al., 2026). Apart from the psychological aspects (anxiety, depression, and impaired social functioning), eating disorders often lead to significant physical health complications, such as electrolyte imbalances, gastrointestinal issues, and cardiovascular problems (Balasundaram & Santhanam, 2026; Ortiz et al., 2026). A range of risk factors has been identified in the development of eating disorders. Biological and genetic vulnerabilities play a significant role, with heritability estimates and neurobiological mechanisms contributing to individual susceptibility (Bulik et al., 2006; Striegel-Moore & Bulik, 2007). These highlight the multifactorial etiology of eating disorders, emphasizing the interaction between biology, cultural and psychological characteristics.

The Global Burden of Disease Study has shown that the incidence of AN and BN is increasing globally (Liu et al., 2025). These statistics are troubling in themselves; however, Santomauro et al. highlighted the potential true burden of eating disorders when they performed an analysis that included BED and OSFEDs in addition to AN and BN. Including all eating disorders significantly impacted the ranking, moving eating disorders to the 73rd cause of disability-adjusted life-years from the 110th cause, with a new total of 55.5 million people affected, a large increase from the 13.6 million with AN and BN only (Santomauro et al., 2021). The number of people affected by eating disorders is increasing, estimated to have doubled from 2000 to 2018 (3.5% to 7.8%) (Galmiche et al., 2019). It is estimated that in the U.S., 9% of the population will be diagnosed with an eating disorder in their lifetime (Deloitte Access Economics, 2020). Between 2000 and 2018, the global lifetime prevalence of the three primary eating disorders were 0.2% for males and 1.4% for females for AN, 0.6% for males and 1.9% for females for BN, and 1.0% for males and 2.8% for females for BED. Of all the disorders, OSFEDs have a much higher global prevalence of 3.6% for males and 4.3% for females (Galmiche et al., 2019). More importantly, the number of people with eating disorders is predicted to increase annually (Liu et al., 2025), especially BED and OSFEDs (Santomauro et al., 2021). There is some evidence to suggest that people with eating disorders have more health issues compared to those without an eating disorder (Cloak & Powers, 2005). In U.S. hospital emergency departments, 15.9% of patients aged 21–65 years screened positive for eating disorder risk (Dooley-Hash et al., 2019). In Italy, people with an eating disorder accessed emergency departments more than twice the rate of matched controls (Castellini et al., 2023). Furthermore, considering all psychiatric disorders, eating disorders are associated with the highest rate of all-cause mortality (Dooley-Hash et al., 2019).

The etiology of eating disorders is complex and has recently been reviewed (Barakat et al., 2023; Bulik et al., 2022; Keski-Rahkonen, 2024; Terry et al., 2022). There is no doubt that weight and body image concerns are risk factors for eating disorders. While these issues have been ever-present in Western culture, excess social media, a relatively new form of cultural communication, may further increase the risk of eating disorders (Dane & Bhatia, 2023), especially with the availability of sites that promote eating disorders (Jett et al., 2010). Less is known regarding the effect of ethnicity. Some evidence suggests that African Americans may have a higher risk of BED, and thin-ideal internalization is more prominent in Asian Americans, compared to White Americans. Similarly, socioeconomic status seems to have little overall effect; however, more education increases the risk of AN, while food insecurity is associated with binge behaviors (Barakat et al., 2023). Temperamental traits, such as perfectionism, negative emotionality, and harm avoidance, have also been consistently associated with increased risk, shaping how individuals respond to environmental stressors (Lilenfeld, 2011). For example, childhood weight, bullying (including cyberbullying), trauma and abuse are associated with risk of eating disorders (Barakat et al., 2023). There are clear gender differences related to risk. Up to 17.9% of young women and 2.4% of young men have reported experiencing an eating disorder; OSFEDs are most common in young women, and BED is more common in young men (Silén & Keski-Rahkonen, 2022). Gender is complex, as puberty, gender roles and sexual identity all impact risk (Barakat et al., 2023). There is also a genetic heritability component to eating disorders. Having a relative with AN means a person is 11 times more likely to develop AN; the risk ranges from 4 to 10 times more likely to develop BN, while the odds of developing BED are approximately double if one has a relative with BED. Interestingly, the behavior associated with intentional weight loss may have genetic foundations (Thornton et al., 2011). Genetic data are strongest for AN and BN; however, some data suggest that night eating syndrome (NES) may also have genetic components (Barakat et al., 2023). In addition to all the above risk factors, there are some emerging risk factors, such as gut microbiome differences, the immune system and comorbidities such as type 2 diabetes in BED (Barakat et al., 2023; Feng et al., 2023), that all play a role in an individual’s risk of developing an eating disorder.

Because eating disorders often emerge in adolescence and young adulthood, university settings are an important time for screening and prevention. University brings some freedom to students, though at a cost, as university students need to be independent. This adds stress and anxiety, from academic and social demands, time management and even part-time work (Worsley et al., 2021). There is also a need to have a sense of belonging to a university to help successfully navigate university life (Stubblebine et al., 2024). A lack of belonging may be related to imposter syndrome, which has a relatively high prevalence in medical students, as well as other healthcare professionals (Salari et al., 2025). Furthermore, risk factors for imposter syndrome and eating disorders overlap. Academic, financial and personal stressors are most frequently reported by university students, which have a significant impact on mental health (Reis et al., 2021). Compared to the general population, the risk of having an eating disorder is higher for university students, estimated at 19.7% (Alhaj et al., 2022). This estimate agrees with other investigators (Falvey et al., 2021; Tavolacci et al., 2015); however, the COVID-19 pandemic may have increased the risk to 51.8% for women (from 31.8%) and 31.3% for men (from 13.3%) (Tavolacci et al., 2021), although this risk elevation has not been confirmed in other studies. Medical students may be particularly at risk of eating disorders because they train in environments marked by intense academic demands, performance pressure, sleep disruption, and chronic stress while also navigating professional identity formation and body image expectations (Alhaj et al., 2022; Grammer et al., 2020; Tavolacci et al., 2021). Eating disorder risk among medical students varies widely across countries and instruments, but the burden is consistently clinically relevant (Fekih-Romdhane et al., 2022; Jahrami et al., 2019b). This variation also suggests that prevalence should be interpreted alongside context, including geography, culture, stage of training, and the screening tool used. A meta-analysis including medical students from nine countries estimated an overall eating disorder risk of 10.4% (Jahrami et al., 2019b), whereas a larger meta-analysis using data from 20 countries estimated the risk of an eating disorder for medical students to be 17.35% (Fekih-Romdhane et al., 2022). In medical students, the prevalence varies extensively based on gender, geography, culture, ethnicity and year, even when utilizing the same instrument (Fekih-Romdhane et al., 2022; Jahrami et al., 2019b). While the overall prevalence in medical students (Fekih-Romdhane et al., 2022) is lower than that in university students in general, the sample size was almost seven times higher in a study of university students that included medical students (Alhaj et al., 2022). A key factor in choosing medical students for this review is that these individuals will be future healthcare providers who may need to diagnose or care for patients with eating disorders.

Eating disorders pose severe physical and psychological consequences (Austin et al., 2021; Berkman et al., 2007; Hambleton et al., 2022; Momen et al., 2021), emphasizing a need for a comprehensive understanding of the factors contributing to their onset and persistence (Pastore et al., 2023). A meta-analysis of efforts to prevent eating disorders in university students showed some success; however, there was low participation from students (Harrer et al., 2020). While each individual study contains some clues to the potential causes, no single factor causes eating disorders. Complex interactions between known risk factors and individual traits, experiences and genetics lead to eating disorders, suggesting each individual has unique causes. This makes the study and prevention of eating disorders challenging. It is possible that some modifiable risk factors may be managed through broad prevention programs; however, many other individual temperament-related risk factors may not.

Therefore, the purpose of this narrative review is to combine the available data to elucidate the key contributors to eating disorder risk in medical school students and offer insights to raise awareness and help in treatment and prevention.

## 2. Materials and Methods

### 2.1. Search Strategy

This is a narrative review focusing on the causes of eating disorder risk in medical students. The sources of literature were PubMed and Google Scholar. The following search phrases were used to find the primary material: “eating disorders medical students” and “eating disorders medical school”. In addition, the authors searched using the “similar article” tool in PubMed, and citation lists in the identified papers were also reviewed for inclusion. Inclusion criteria included: article in English or Spanish, prevalence data for medical students, sample size and gender, and data related to possible causes of eating disorder risk. There were no restrictions on dates to find as many studies as possible. All articles that had medical students among the participants were included, but only articles specific to medical students, with a recognized eating disorder screening instrument, were included in the table. All validated eating disorder risk instruments were included.

In addition to searching for the primary material, literature searches were performed for supporting material and consisted of combinations of “eating disorders”, “university students”, medical students”, “physician”, “physical activity”, “sleep”, “bone health”, “social media”, “chronic stress”, “stress”, “epigenetics”, “neuroplasticity”, “genetics”, “temperament”, “nutrition”, “diet” and “mindfulness”.

### 2.2. Data Use and Analysis

The data from published studies were compiled into a descriptive table. No data analysis or transformation of data was performed. For factors contributing to eating disorders in medical students, the risk factor data were classified into four major themes.

## 3. Results

### 3.1. Prevalence of Eating Disorders

Investigating the reasons for the increased prevalence of eating disorders in medical students is important, as it may affect their professional lives and may introduce bias in their encounters with patients with eating disorders. The prevalence of eating disorders in medical students and significant risk factors for eating disorders from different countries are summarized in Table 1.

Overall, the predominant instrument used is the Eating Attitudes Test-26 Item (EAT-26). The EAT-26 was developed in the 1980s as a shorter version of the EAT-40 (Garner et al., 1982) to help rapidly screen for eating disorders (McLean et al., 2023). It consists of 26 items related to one’s attitudes and behaviors towards food. However, it may have limitations related to culture (Spivak-Lavi et al., 2023) and vegans (McLean et al., 2023). This may explain the large ranges; however, all instruments have limitations. The SCOFF (Sick, Control, One, Fat, Food) questionnaire is a series of five questions designed to illustrate core experiences felt by individuals with eating disorders. Even though this questionnaire has been shown to be effective in the screening of eating disorders, further research may still be needed to establish its validity and reliability in the general population (Morgan et al., 2000).

Prevalence for male and female medical students ranges from as low as 9.5% (Iran) to 36.76% (Egypt), with both using the EAT-26 as the screening instrument. Bahrain, Chile, Egypt, Saudi Arabia, Lebanon, Pakistan, and Peru showed the highest amount of risk for the development of an eating disorder among medical students, with EAT-26 scores higher than 20%. The meta-analysis by Fekih-Romdhane et al. found that medical students in Austria and Hungary had the lowest risk, while risk was highest for Lebanese medical students (Fekih-Romdhane et al., 2022). This agrees with the general trend that Westernization of countries increases eating disorder risk (Liu et al., 2025), and this has also been seen in medical students (Fekih-Romdhane et al., 2022). Comparing gender differences using EAT-26, the prevalence ranged from 2.61% (Brazil) to 35.5% (Peru) for males, while for females, the prevalence ranged from 7.5% (India) to 85.3% (Egypt). In the U.S., EAT-26 found that 14.5% (n = 69) of medical students were at risk of an eating disorder, and SCOFF found that 25.6% were at risk (Cummings & Uhley, 2024). Using a questionnaire designed for the study by the authors, out of 121 female U.S. medical students, 4.1% met the criteria for bulimia, 3.3% reported a history of anorexia nervosa and 8.3% historically met the criteria for bulimia (Herzog et al., 1985). The Moroccan study (Azzouzi et al., 2019) does not specify which instrument was used for the reported prevalence; however, it may likely have been SCOFF. The data show that overall, female medical students may be at a higher risk; however, in a few studies, male and female prevalence are relatively close (Belhaj Salem et al., 2025; Carpio-Oviedo et al., 2025; Iyer & Shriraam, 2021; Naeimi et al., 2016; Ngan et al., 2017), suggesting that cultural factors or professional identity may play a role. Only one study included a specific non-binary medical student analysis, and the prevalence was 26% (Tavolacci et al., 2025). While this value falls in the range for female medical students, more data are required to investigate if the risk is even higher in medical students of sexual and gender minorities.

Overall, the ages of participants in the studies were similar. One study found that being aged between 22 and 25 years was associated with a higher risk of having an eating disorder (Bizri et al., 2020), while another found that medical students aged 20 years or older had a higher risk (Alberton et al., 2013). For both these studies, the majority of participants were in the age groups associated with eating disorders, at 84% and 70%, respectively. There was a relatively narrow age range across all studies, suggesting the sample size may not be sufficient to use age as a variable.

### 3.2. Factors Contributing to Eating Disorders in Medical Students

While this review aims to compile risk factors (statistically significant) for eating disorders in medical students, there are already inherent behavioral risk factors within the screening tools that must be considered. EAT-26 contains questions related to restrictive and binge eating, e.g., “Am terrified about being overweight”, “Am preoccupied with the thought of having fat on my body” and “Like my stomach to be empty”, and questions on laxative, diet pill and diuretic use, as well as exercise to lose weight and recent weight loss. The SCOFF questionnaire is similar, e.g., “Do you make yourself sick because you feel uncomfortably full?”, Do you believe yourself to be fat when others say you are too thin?” and “Would you say that food dominates your life?”, and includes a recent weight loss and an appetite control question. Therefore, when using these questionnaires, individuals who are at risk already possess several of these risk factors. Several studies document sections of EAT-26 or Eating Disorder Inventory second edition (EDI-2) (Alberton et al., 2013; Aljaffer et al., 2026; Azzouzi et al., 2019; Bizri et al., 2020; Elsherif et al., 2025; Ghamri et al., 2022; Mallaram et al., 2023; Mandiola et al., 2022; Memon et al., 2012; Sfeir et al., 2023); however, it is heterogenous. This suggests a need to consistently report significant behaviors from screening questionnaires or include individual responses in future analyses, which may help elucidate synergies between behaviors and pathophysiology, especially in the age of machine learning.

In addition, among the studies included in Table 1, a host of questions are used investigate the risk factors of eating disorders. These include academics, psychological health (e.g., stress, anxiety, depression), alcohol, smoking, skipping meals, body image, self-esteem, physical activity, living situation, sleep, mental health history, country of origin, family history, social media use, finances, COVID-19 pandemic, year of medical school, marital status, sexual activity, sexual assault/harassment, healthcare usage and parents’ education level, among others. The majority of these did not make a statistically significant contribution to eating disorder risk, so they were not included here. However, given the heterogenicity in the studies and the complex etiology of eating disorders, these factors may have some impact on the sociocultural model, but larger sample sizes and computational statistics will be required to reveal interactions between risk factors.

With this in mind, for this review the inherent behaviors within the screening questionnaires will not be included; however, it is appropriate to consider that those at risk already displayed recognized behaviors for restrictive and binge/purge eating disorders. Furthermore, the variables that were not statistically significant within the studies will not be included; however, many of these variables contribute to shaping an individual’s life experience and thus contribute to trait and temperament, suggesting these should not be ruled out in future studies. Based on the studies in Table 1, there are four major themes that contribute to eating disorders in medical students: (1) body image and weight loss, (2) regular physical activity, (3) curriculum and (4) psychological well-being.

#### 3.2.1. Body Image and Weight Loss

The COVID-19 pandemic has certainly had an impact on body shape (Almahmeed et al., 2025); medical students noted worse eating habits, while 51.1% of students became more preoccupied with body image and weight. However, its effects may phase out over time. Body image concerns in medical students preceded the COVID-19 pandemic, although the pandemic may have provided an environment that compounded them. A meta-analysis including 21,383 participants from twenty countries found that increased BMI was the only statistically significant predictor of eating disorder risk (Fekih-Romdhane et al., 2022). However, a study in India found that BMI was not positively associated with a higher risk of eating disorders (Iyer & Shriraam, 2021). Studies from Brazil showed that the desire for thinness and a higher BMI contributed to eating disorder risk (Alberton et al., 2013; Bosi et al., 2016). Body shape concerns (using the Body Shape Questionnaire (BSQ) (Cooper et al., 1987)) were highly positively correlated with higher EAT-26 scores in Indian medical students (Iyer & Shriraam, 2021). Daily exposure to the thin ideal was a significant risk factor for eating disorders in a cross-sectional analysis of 12 countries (Hammad et al., 2025). In the U.S., medical students at risk of eating disorders exhibited weight bias internalization (Hammad et al., 2025), suggesting professional expectations in weight-centric healthcare (Gomez, 2024) may be a factor. Dieting, fasting-induced vomiting and the use of appetite stimulants, all behaviors related to body image, were associated with a higher risk of an eating disorder in Moroccan medical students (Azzouzi et al., 2019). Self-induced vomiting and the use of laxatives were associated with a higher risk of an eating disorder in Indian female medical students (Mallaram et al., 2023). The drive for thinness contributed to Romanian medical students’ risk of eating disorders (Motorga et al., 2025). This is supported by evidence from an earlier Indian study on university students, including medical students, who frequently used laxatives or diuretics (Vijayalakshmi et al., 2017). Female Spanish university students in healthcare-related fields were more image-conscious, fearful of gaining weight, and prone to developing eating disorders than males (Castelao-Naval et al., 2019), suggesting there may be body image expectations for these in health-related fields.

An early study on eating disorder risk in the U.S. consisted of 44% female medical students (n = 96). The mean BMI was 20.7 kg/m^2^, with the highest BMI being 23.6 kg/m^2^, suggesting that all females would be considered healthy weight today. However, using EDI-1, 18.7% of female medical students were within the anorectic range in the drive for thinness scale, and 15.4% were considered bulimic (Futch et al., 1988). In contrast, a higher BMI elevated the odds of being at risk of an eating disorder in Egyptian medical students (Ali & Shehata, 2020; Elsherif et al., 2025). Interestingly, in Hungarian medical students, there was a significant increase in compensatory behaviors to avoid weight gain in males (8.9% to 14.6%) over 12 years, but not in females, suggesting a cultural shift in male body type expectations (Tury et al., 2020). In Danish medical students, avoidance of fatty foods was common among males and females; however, only 1.9% of males reported going on a diet, compared to 16.9% of females (Dissing et al., 2011). In Indian female medical students, weight loss ≥ 9 kg in the past six months was associated with a higher risk of an eating disorder (Ghamri et al., 2022). Being overweight or obese resulted in an up to 2.69-fold adjusted odds ratio of being at risk of an eating disorder in medical students at a medical school in the Kingdom of Saudi Arabia (Ghamri et al., 2022). Conversely, weight loss ≥ 9 kg in the past six months was not associated with an increased risk of eating disorder risk in Lebanese medical students (Bizri et al., 2020), suggesting it may be more culturally acceptable. While only 10.6% of students in one study were considered obese (by BMI), obesity had almost a four-fold increased odds (OR = 3.9) of being at risk of an eating disorder in medical students studying in Malaysia (Ngan et al., 2017). Parenting has a large impact on children’s development and personality. Having a university-educated mother was a risk factor for eating disorders in Iranian medical students (Naeimi et al., 2016). Parental education is a well-known risk factor for eating disorders, possibly due to perceived higher expectations (Sawma et al., 2025). Daughters with higher self-esteem and better overall psychological health were at a lower risk of poor dietary behaviors (Spivak-Lavi et al., 2024). In addition, the risk of an eating disorder is decreased if mothers have a positive body image of themselves and promote healthy beliefs about body image to their daughters (Handford et al., 2018). These data support the premise that body image and BMI, which are interrelated, are contributors to eating disorder risk in medical students.

#### 3.2.2. Regular Physical Activity

Physical activity and exercise (a specific type of physical activity (Oppert et al., 2023)) are related to body image (Hausenblas & Downs, 2001; Hausenblas & Fallon, 2006), especially muscularity in males (Piatkowski et al., 2024) and the disengagement from exercise in females (Matheson et al., 2025), and exercise is a component of weight loss (Oppert et al., 2023). However, for this review, physical activity is separated from body image and weight loss as it is a central element of a healthy lifestyle, with many physical and psychological benefits, such as preventing chronic disease and healthy aging (Dhuli et al., 2022), and is not limited to improving body image or being a requirement for weight loss. Regular exercise was found to be a protective factor (reduced the risk of eating disorders) for medical students (Belhaj Salem et al., 2025). Exercise is a much more complex factor due to its potential to also contribute to eating disorders (Godoy-Izquierdo et al., 2023). Excessive exercise occurs when individuals engage in compulsive physical activity to control weight, shape, or feelings of guilt related to eating. In contrast, the lack of regular physical activity may play a significant role in increasing the likelihood of developing an eating disorder. Regular physical activity has definite physical and psychological benefits (Gerber et al., 2025), while overtraining can have various negative physical consequences, but more importantly, there can be a psychological addiction to exercise (Adams & Kirkby, 2001; Adams & Kirkby, 2002). Therefore, the duration and intensity of exercise may be a factor to consider in future studies. In Egypt, medical students were 1.75 times as likely to have an eating disorder if they did not participate in regular physical activity, and being overweight or obese (classified using BMI) elevated the odds of being at risk of an eating disorder (Ali & Shehata, 2020). A larger study in Egypt found that 54% of medical students were of a healthy weight, and lack of physical activity was associated with being overweight or obese (Nowara et al., 2025). A cross-sectional study in the Middle East and North Africa (MENA) region (Palestine, Egypt, Syria, Tunisia, Morocco, Sudan, United Arab Emirates (UAE), Jordan, Algeria, Libya, Kingdom of Saudi Arabia and Pakistan) found that regular physical activity and body weight satisfaction were associated with a lower risk of an eating disorder (Belhaj Salem et al., 2025). Regular exercise (compared to daily rigorous or zero exercise) in Indian medical students was associated with a lower risk of eating disorder (Iyer & Shriraam, 2021). Physical fitness declined in Chinese medical students over the course of medical school (Yi et al., 2019). Physical fitness relieves stress (Childs & de Wit, 2014; Mucke et al., 2018); however, it is not known what level is needed. Interestingly, ancient Greek schools integrated physical activity with learning (Fraile-Martinez et al., 2025). Mental distress was associated with a lack of physical activity in medical students and decreased resilience (Azim et al., 2025), showing the beneficial effects of physical activity on stress in medical students. Exercise is recognized as a key method to alleviate stress in university students, including medical students (Guerriero et al., 2025), especially mindfulness activities such as yoga (Chauhan et al., 2024). These data suggest that student wellness programs should target moderate exercise and mindfulness for chronic stress relief.

#### 3.2.3. Curriculum

Five studies indicate that medical school curricula may have an impact on eating disorder risk. Medical students had a lower risk of an eating disorder during the clinical years of medical school in Bahrain (Almahmeed et al., 2025). Brazilian medical students had 1.7 times the prevalence of the risk of an eating disorder in the first five semesters of their education compared to those in semesters 6–10 (Alberton et al., 2013). French medical students had a reduced risk of an eating disorder in the clinical years and into residency (Tavolacci et al., 2025). Similarly, in the Kingdom of Saudi Arabia, those in the preclinical stage of medical education had 1.77 times increased odds of being at risk of an eating disorder (Ghamri et al., 2022). Conversely, Bizri et al. found no association between risk of eating disorders and year of study (Bizri et al., 2020). Although the effect size was small, fewer medical students were at risk in a Norwegian medical school using a problem-based curriculum compared to a standard curriculum, with values of 20.6% and 16.0%, respectively (Rostad et al., 2021). These data show that the preclinical curriculum is the most stressful; this stress may be related to preparing for the first medical board exam, as it is in the U.S., which must be passed before beginning clinical training. These data suggest that patient interactions in the clinical years may help students realize their professional identities and reduce stress. This conclusion is somewhat supported by the Norwegian study, which suggests problem-based learning may be a better strategy for the preclinical years because it is more patient/clinical-centric.

#### 3.2.4. Psychological Wellness

Eating disorders are psychiatric illnesses, and some psychological factors are expected. Schizophrenia, autism spectrum disorder, food insecurity and less sleep were associated with a higher risk of eating disorders in a cross-sectional study of 12 countries in the MENA region (Belhaj Salem et al., 2025). In Bahrain, 68.8% of medical students reported mental health issues, and students who were living alone or with families, or who were not local, were at a higher risk of an eating disorder (Almahmeed et al., 2025). Female Chilean medical students with higher personal standards and higher perceived stress had 16% and 9% higher odds of an eating disorder, respectively, based on a multivariate analysis (Mandiola et al., 2022). Probable alexithymia was found to increase risk in Egyptian final year medical students (Elsherif et al., 2025). In French medical students, depression, anxiety and suicidal ideation were all associated with those at risk of an eating disorder, as was sexual harassment and forgoing psychotherapy for financial reasons. Additionally, more French medical students who were at risk of an eating disorder considered dropping out of medical school (Tavolacci et al., 2025). In the U.S., 70% of those at risk did not receive mental health treatment (Cummings & Uhley, 2024), which may be due to financial reasons; however, this was not investigated. Existing self-reported psychiatric illnesses and exhibiting eating disorder behaviors, such as binge eating, were associated with a higher risk of an eating disorder in Indian female medical students (Mallaram et al., 2023). In contrast, medical students from the United Arab Emirates reported consuming less food when stressed (Nowara et al., 2025). For Iranian medical students, the odds of being at risk of an eating disorder were 63% lower for those with good self-esteem (Naeimi et al., 2016), suggesting self-esteem may be a good target because stress and self-esteem are interrelated, as are self-esteem and psychological health (Abouserie, 1994; Galanakis et al., 2016; Kinariwala et al., 2025; Nahar et al., 2025). A reported history of mental health diagnoses put Lebanese medical students at a higher risk of an eating disorder. However, a family history of eating disorders was statistically significant for EAT-26 (*p* = 0.038) but not SCOFF (*p* = 0.062) (Bizri et al., 2020). At a private medical institution in Malaysia, not being satisfied with friends was significantly associated with eating disorder risk. While 41% of the students were Malay, there was a tendency (*p* = 0.062) for Indian students to be at more risk of an eating disorder (Ngan et al., 2017). Stress significantly increased the risk of an eating disorder in Norwegian medical school students (Rostad et al., 2021), while there was only a moderate association (Cramer’s V coefficient = 0.38) between the risk of an eating disorder and experiencing moderate or severe stress in Peruvian medical students after returning to in-person training after the COVID-19 pandemic (Carpio-Oviedo et al., 2025).

Stress impacts the psychological health of medical students (Haykal et al., 2022). However, only four studies investigated stress, especially academic stress, which did contribute to eating disorder risk; see Table 1 (Bizri et al., 2020; Carpio-Oviedo et al., 2025; Mandiola et al., 2022; Rostad et al., 2021). This may be because stress is inherent in medical school (Hawsawi et al., 2025); however, medical school stressors need to be studied in the context of eating disorder risk. In a study in Iraq, almost 21% of the medical students included in the analysis were at risk of an eating disorder, with stress as the main contributing factor (Shanshal et al., 2022). Having unsatisfactory social relationships with friends, as seen in the study on Malaysian medical students, was a risk factor for eating disorders (Ngan et al., 2017). In general, people without supportive friends are more likely to have depression and an unfulfilled life (Choi et al., 2020), as well as lower mortality (Holt-Lunstad et al., 2010). Medical students with robust social networks during medical school may have enhanced learning (Vaughan et al., 2015; Woolf et al., 2012). Supportive friends may also help students cope with medical school stress (Sajid et al., 2024; Salih et al., 2023), which may help prevent eating disorders. Approximately three-quarters of medical students (73.4%) experience moderate (29.1%) or severe (44.3%) symptoms of post-traumatic stress disorder (PTSD) (Habib et al., 2022). Additionally, female medical students and students from mixed rural/urban backgrounds have a higher prevalence of PTSD (Ahmad et al., 2018). With the rates of PTSD being so high amongst medical students, it is important to consider the implications that this type of stress induces. Apart from a lack of emotional regulation, both PTSD and eating disorder patients exhibit a heightened incidence of anxiety disorders—the most common class of psychological conditions that are highly comorbid with other disorders (Williamson et al., 2021). Studies have shown that PTSD can lead to the development of an anxiety disorder and that anxiety disorders predispose individuals to developing eating disorders (Swinbourne & Touyz, 2007). Among medical students, symptoms of anxiety disorder range between 7.7% to 65.5%, with female medical students showing more symptoms than male students (Mirza et al., 2021). Therefore, it is possible that the high prevalence of anxiety disorders predisposes students to PTSD and puts this population at a higher risk of developing an eating disorder during their professional career.

## 4. Limitations and Future Needs

### 4.1. Study Heterogenicity

There are several limitations to the data discussed here. Data heterogenicity was discussed in previous meta-analyses (Fekih-Romdhane et al., 2022; Jahrami et al., 2019b) and in a cross-sectional study (Belhaj Salem et al., 2025). However, the majority of the studies used the EAT-26 instrument, which made comparing the studies easier. While there is great heterogenicity, it may be perceived as necessary to capture risk factors among different cultures; however, there were no overtly different questions that could not be applied to all cultures. Overall, there is an urgent need for longitudinal studies and interventional trials related to eating disorders in medical students.

### 4.2. Unusual Findings

Some of the statistically significant risk factors were difficult to categorize. These included not being married, living alone or with family, having breakfast more than half the time in a week and >1 current sexual partners; see Table 1. Not being married was associated with eating disorder risk in Egyptian medical students (Ali & Shehata, 2020). In general, being single is associated with a higher mortality than being married (Jia & Lubetkin, 2020), and there may be less stress in married people compared to single people because of the support structure (Gavalas, 2025). The finding that being married elevates eating disorder risk in medical students seems contradictory; however, married medical students may experience more stress or other issues due to family and spouse commitments. Furthermore, because the majority of participants were single, there is not enough data to say with any certainty that being single is a risk factor for eating disorders in medical students. Therefore, the question of the effect of marriage on medical student wellness remains to be answered. In Peruvian medical students, having breakfast more than half the time in a week was associated with eating disorder risk (Carpio-Oviedo et al., 2025). Skipping meals is typically considered poor dietary behavior and is associated with depression and stress (Pendergast et al., 2016; Tajik et al., 2016). The question in the study was worded so both options included skipping breakfast; the nuance was in the number of times breakfast was skipped (Carpio-Oviedo et al., 2025). Since the finding suggested that those who skipped fewer breakfasts were at a higher risk, the results are again somewhat contradictory; however, other contexts around breakfast may be important, such as eating breakfast alone versus with friends. It is important for future studies to assess nutritional behaviors in more detail. In Ugandan medical students, having more than one sexual partner was associated with a more than fourfold increase in the odds of risk for eating disorders (Abaatyo et al., 2025). This may be related to low self-esteem, emotional instability and impulsive/risky behaviors, as well as poor body image, as the authors explained. Adolescents with sexual partners of both sexes have elevated odds of eating disorder behaviors (Ackard et al., 2008; Yu et al., 2018). The same risky sexual behaviors are seen in young adult women with eating disorders (Fergus et al., 2019). Sexual activity may be an important consideration for future studies. Being the first-born child was a statistically significant risk factor for eating disorder risk in Egyptian final-year medical students (Elsherif et al., 2025). This is an interesting finding, as there may be some pressure related to being the eldest child; however, there is a major lack of data related to this aspect for medical students.

In the Kingdom of Bahrain, living alone or with family was associated with eating disorder risk (Almahmeed et al., 2025). Both living situations have different possible explanations for contributing to or even reducing eating disorder risk (living alone can mean less support, but it can also mean fewer distractions from study, and living at home adds family dynamics, which can be negative or positive). Living conditions can be complex, and more details are needed to address this point; however, it will be a great addition to future studies.

### 4.3. Reliance on BMI as a Variable

High BMI was recognized as a risk factor in several studies; however, BMI can be a physiological and cultural limitation. BMI is a proxy measure for fat mass in large epidemiological datasets and has well-described limitations in relation to measuring fat mass or lean mass in individuals (Ilich, 2020; Potter et al., 2025). Furthermore, normal-weight obesity, also known as thin-outside–fat-inside, metabolically obese normal weight, and colloquially as “skinny fat”, describes an individual who has a healthy BMI yet is obese due to the presence of excess body fat (Oliveros et al., 2014). The use of BMI has limited the nature of obesity in individuals, and there are many phenotypes of obesity that body composition can help distinguish (Yarnoz-Esquiroz et al., 2022). People with normal-weight obesity have the same risks of cardiovascular disease and mortality (Batsis et al., 2013; Mohammadian Khonsari et al., 2022), functional disability in older females (Batsis et al., 2014) and type 2 diabetes (Bai et al., 2024; Kapoor et al., 2020) as those with a high BMI. The issue is that these people would not receive targeted medical care for adiposity reduction and diabetes prevention, as they would be perceived as “healthy” based on their good BMI. The other limitation of BMI in terms of health is the concept of metabolically healthy obese (high BMI with healthy blood pressure, triacylglycerides, HDL and fasting plasma glucose) (C. Tanriover et al., 2023). The metabolically healthy obese and metabolically unhealthy obese phenotypes have been extensively reviewed (e.g., Bardanzellu et al., 2020; Bosch-Sierra et al., 2024; Iacobini et al., 2019; Juarez-Fernandez et al., 2021). BMI is used clinically as it is rapid, inexpensive and requires few resources. In 2023, the American Medical Association recommended that BMI should no longer be the sole or main measure for diagnosing obesity at the individual level (Berg, 2023; Tanne, 2023). This is especially important because eating disorder risk factors and treatment need to be considered at the individual level. Preliminary studies show no association between body fat percentage and eating disorder risk in athletes (Magee et al., 2023). However, in Slovakian adolescents, those with a higher body fat percentage were associated with eating disorder risk (Stefanova et al., 2020). Regardless, there is no doubt that body composition has a future in monitoring eating disorder treatments, as the goals would include increasing lean mass (Dalili et al., 2020). The limitations of BMI are well described in ethnic and racial minorities, rooted in its origins in Northern European cohort databases (Bays et al., 2022; Pray et al., 2023; Rao et al., 2015). This suggests body composition measures are needed for future eating disorder studies, especially in multiracial studies, as they may help determine physiological risk factors.

### 4.4. Gender and Sexual Minority Gaps

There is a major limitation in relation to gender differences. For male medical students, data are limited (Jahrami et al., 2019b). Most studies on medical students find that females are at a higher risk. As the sample size of males is much smaller, further stratification for different variables may not be possible with current data. Other screening tools specific to males may be needed, for example, the Muscularity Oriented Eating Test (Murray et al., 2019), as male body dissatisfaction seems to be increasing (Ralph-Nearman & Filik, 2018). There is growing concern regarding eating disorder risk in gender and sexual minorities. This population may be uniquely positioned to be at the greatest risk of an eating disorder, as it was estimated that half will receive a diagnosis of an eating disorder during their lifetime (Nagata et al., 2024). The U.S. Healthy Minds Study showed that among undergraduate and graduate university students, higher numbers of questioning and bisexual men and women were at risk of an eating disorder compared to heterosexual men and women. Gay men and bisexual and lesbian women were also at higher risk (Hazzard et al., 2020). To our knowledge, only one study on medical students included non-binary individuals, and their prevalence was higher than males but lower than females (Tavolacci et al., 2025). Future studies will need to address these gender and sexual minority gaps.

### 4.5. Chronic Stress

A key gap is the lack of data related to the effects of chronic stress in medical students. While acute stress is concerning, chronic stress is the key issue (Shchaslyvyi et al., 2024). Regular exams, academic and community expectations, professional conduct expectations and developing professional identity are constant forces in medical school. This chronic high stress is consistently reported globally among medical students (Jahrami et al., 2023). Medical students, who are often immersed in a mentally rigorous and competitive environment, commonly exhibit chronically higher levels of stress, leading to a greater risk of adverse effects (Neufeld-Kroszynski et al., 2024). The day-to-day academic demands and the challenge of clinical rotations can affect the psychological well-being of medical students (Perni et al., 2020). Additionally, the high incidence of reported levels of stress among medical students is linked to excessive workloads, time management difficulties, conflicts in work–life balance, health distress, and stress due to financial debt (M. R. Hill et al., 2018). Medical students consistently report higher levels of perceived stress, academic stress, financial stress, moral distress, PTSD, and anxiety disorders compared to the average population (Heinen et al., 2017; M. R. Hill et al., 2018). While medical students described exams as a high stressor, female medical students perceived their physical health status and general stress level to be worse when compared to male students (Backovic et al., 2012). Additionally, the prevalence of burnout in medical students was found to range from 5.6% to 88%, with females being at a significantly greater risk of burnout syndrome than males (Di Vincenzo et al., 2024), suggesting gender differences. Chronic exposure to high levels of stress can have negative effects on both the physical and psychological health of medical students, specifically at the level of cognitive functioning, ability to sleep, and overall academic performance (Almojali et al., 2017). Furthermore, imposter syndrome is common among medical students, which significantly impacts academic stress (Khan, 2021). Academic stress may be a risk factor for stress-undereating (Emond et al., 2016), which could contribute to malnutrition and more negative health complications. Malnutrition can induce additional epigenetic changes (Ortega et al., 2022). Overtime, the interactions between stress, diet behaviors and epigenetics will result in a positively reenforced cycle sustaining the development of an eating disorder (Steiger & Thaler, 2016). This phenomenon helps explain why malnutrition in anorexia nervosa patients serves as an added stressor that contributes to the phenotype (Chami et al., 2019).

Furthermore, the relationship between chronic stress and its repercussions on the physiological and psychological well-being of individuals remains a subject of great importance (Cohen et al., 2007) that needs to be explored. The intricate relationship between different types of stress and their impacts on epigenetics (the regulation of gene expression without changing an individual’s DNA sequence) and neuroplasticity in individuals has emerged as a critical platform for understanding the potential risk factors for eating disorders (Bin Mugren & Al Turki, 2021; Frank et al., 2019; Hardaway et al., 2015; Wierenga et al., 2018). There is an urgent need to address chronic stress in medical school students, and new instruments may need to be developed.

A distinct neuroplastic change is seen in the functional connectivity of the brains of individuals with eating disorders, such as AN, BN, and avoidant/restrictive food intake disorders (AFRIDs), compared to healthy controls. In addition, many complex networks are completely altered, such as the default mode network (DMN)—responsible for the perception of sensations (sixth sense): the salience network—which helps orient healthy food intake as a positive stimulus, and the reward-processing/decision-making network—which is crucial in facilitating addictive behaviors (Frank et al., 2019). Individuals with eating disorders tend to showcase increased connectivity in the DMN, which correlates with increased self-rumination, as seen with individuals suffering from depression. Furthermore, an elevated DMN can potentially underscore the brain regions responsible for driving the persistent self-rumination and cyclical nature of both diseases (Chou et al., 2023; Frank et al., 2019). Additionally, the salience network showcases a significant positive correlation with body shape questionnaire scores, indicating that these individuals regard body image as a stimulus of much higher relevance compared to healthy controls (Frank et al., 2019). Lastly, the reward-processing/decision-making network is shown to have much stronger connections between the ventral striatum and frontal cortex, which is a crucial similarity shared among many addictive behaviors (Frank et al., 2019; Koob & Volkow, 2016). The ventral striatum is responsible for the initiation of habitual behaviors and is hypothesized to underlie compulsive responses to drugs (Koob & Volkow, 2016). The elevated connectivity of the ventral striatum reinforces the addictive nature behind the behaviors seen in people with eating disorders. These three networks, DMN, SN, and reward-processing/decision-making, which are changed in people with eating disorders, are similarly altered by chronic high stress exposure. In addition, those with higher perceived stress showed increased functional connectivity of the DMN, a drastic change in functional connectivity of the SN, and complete modulation of the reward-processing network (Baik, 2020; Soares et al., 2017; Zhang et al., 2022). The evidence suggesting that high stress can lead to similar neuroplastic changes seen in people with eating disorders highlights how exposure to high stress can be an influential risk factor for medical students to become more susceptible to eating disorders. Childhood stress and especially post-traumatic stress disorder (PTSD) can have lifelong consequences for an individual’s mental health. With the rates of PTSD being high amongst medical students, it is important to consider the implications that this type of stress has on eating disorder risk for medical students.

### 4.6. Comparisons Between Academic Fields

The data show that medical students are at a higher risk compared to the average risk, due to a unique combination of high levels of stress, perfectionism, disrupted sleep, frequent evaluations, and exposure to weight- and health-focused training. Furthermore, medical students’ personal experiences with eating disorders may shape their future attitudes toward diagnosing, managing, and counseling patients with these conditions. However, students in other health related fields, as well as university students in general, may be at equal or higher risk. This is a significant gap in the literature, as students in other academic fields may be at higher risk. When compared to nursing students, medical students had a similar risk of eating disorders, with values of 14% and 15%, respectively (O. Tanriover et al., 2025). In Romania, drive for thinness (using EDI-3) was higher in medical students compared to dental and midwife students (9.4% vs. 6.1% and 3.6%), while the bulimia scale was similar in medical and dental students (12.8% vs. 12.2%) (Motorga et al., 2025). Among Indian medical, health sciences, pharmacy and nursing students, medical students were at the highest risk; however, there was only a difference of 3.56% between the highest (medical students) and lowest group (nursing students) (Iyer & Shriraam, 2021). These findings suggests that healthcare education may be a higher-stress environment for students. A meta-analysis of university students in Brazil found that nutritional students had the highest risk of an eating disorder (26%), compared to the risk for all other academic disciplines, including medical students (20.5%) (Stankiewicz et al., 2013). One possible explanation is that nutrition students are trained to think about food more than other disciplines. This academic focus on food may be picked up in the questions in EAT-26, which was the most common instrument. For example, EAT-26 contains the questions: “Find myself preoccupied with food”, “Aware of the calorie content of foods that I eat”, “Particularly avoid food with a high carbohydrate content (i.e., bread, rice, potatoes, etc.)” and, especially, “Give too much time and thought to food”. These are all areas that nutrition students would be trained to be aware of when considering food choices. In addition, the question “Avoid foods with sugar in them” and “Eat diet foods” has become part of general dietary advice. In the Kingdom of Saudi Arabia, eating disorder risk in university students from several colleges were compared. Medical students had the lowest prevalence compared to science students, who had the highest, followed by community medicine and interior design (Alwosaifer et al., 2018). While medical students have a higher risk of eating disorders, other academic disciplines may be catching up or may have been overlooked. This may be due to university becoming more stressful for all students, especially financial concerns (Moore et al., 2021); some university students have part-time jobs (Kroupova et al., 2024). Similar to nutritional students, students in health fields may be more self-aware of energy intake and food choices, and wellness in general, which could skew their screening results. This suggests a need for broader investigations into eating disorder risk across all university academic disciplines.

### 4.7. Genetics and Temperament

One major factor in eating disorder risk is temperament, which was not assessed in any of the studies on medical school students. Because of the heterogenicity in risk factors associated with eating disorders in medical students, it may be beneficial to consider genetic vulnerabilities. Interestingly, there is a high level of genetic overlap in psychiatric disorders (Spivak-Lavi et al., 2023). Recent genome-wide association studies (GWASs) have identified multiple loci associated with eating disorders and revealed genetic correlations spanning both psychiatric domains (e.g., anxiety, obsessive–compulsive traits) and metabolic or anthropometric traits (e.g., insulin sensitivity, lipid metabolism) (McLean et al., 2023). Temperament is the biological basis of one’s personality, including genetics, neural networks and traits (L. Hill, 2024), ultimately driving decision-making. Eating disorders are increasingly understood as having biological underpinnings, which shape sensitivity and response to environmental exposures and stressors. Advances in genomics, neuroscience, and epidemiology support this framework, particularly for AN but increasingly across the full eating disorder spectrum (Bogár et al., 2024; Jäger, 2024; Miller & Pumariega, 2001). Genetic susceptibility is expressed at the neurobiological level through alterations in brain circuits governing reward learning, cognitive control, interoception, and stress responsivity. Therefore, neurobiological differences can lead to altered traits that determine outcomes from external stressors (Bulik et al., 2022). Temperament-driven behaviors, such as dietary restraint or compulsive exercise, can further alter biological systems through starvation, reinforcement of habits, or reward neuroadaptation, creating self-perpetuating feedback loops (Huckins et al., 2024). Recognizing eating disorders as individualized temperament-based (including genetics) disorders may help explain the lack of consistent risk factors in medical school students.

Family, twin, and population-based studies demonstrate moderate to high heritability, particularly for AN. The external trait “sensitivity to stress and adversity” was positively genetically correlated with the following five factors: compulsive, neurodevelopment, internalizing, schizophrenia and bipolar disorders, and substance use disorders. The external trait “hours of exercise” was positively genetically correlated with the following three factors: compulsive, internalizing, and substance use disorders (Jayasinghe et al., 2025; Monterrosa et al., 2020). Based on the findings of the studies with medical students, both of these traits, sensitivity to stress and hours of exercise, may be present in some medical students. However, there may be common traits in medical students, e.g., reason for wanting to go to medical school, and subtle differences in these traits may lead to altered chronic responses, contributing to the risk of eating disorders. Future studies will need to integrate genetic, neurobiological, and temperamental evidence to provide a more precise foundation for understanding the elevated risk of eating disorders in medical school students compared to other academic disciplines.

### 4.8. Food/Diet Culture

While eating disorders are behavioral and not a nutritional issue (unless malnutrition is present), food/diet culture is not explored in depth in eating disorder studies. Food choices are shaped by sociocultural influences (Bogár et al., 2024). Culture influences various aspects of food, including meal timing, preparation methods and cultural prohibitions (Almoraie et al., 2025; Olagunju et al., 2024; Olatona et al., 2020). Cultural food practices have evolved geographically and describe various factors, from which foods are farmed to food practices, such as food manners and timing of meals (Monterrosa et al., 2020). Food is not just a source of energy and nutrients, it is an identity (Fischler, 1988), with Visser stating, “if you change the food you change the culture” (Visser, 1999). In Western culture, food choice is complex. Food/diet is strongly connected to health and weight management, as evidenced by public health messaging, dietary guidelines and commercialization of “diet” products. Furthermore, food choice is becoming more complex as foods need to be sustainable with recyclable packaging, effectively compounding the food decision process already present in Western culture. This may help explain why non-Western cultures adopt thin ideals when integrating Western culture (Pike & Dunne, 2015). Herbs and spices are integrated into traditional Arabic medicine, which has shaped modern Arabic cuisine, while the tradition of fasting is thought to promote health (AlRawi & Zick, 2025). While Arabic food is rooted in health, as is Western food, there is not the same emphasis on thinness. A cluster analysis of global food cultures found nine distinct groups, with Turkey, Japan and Ghana being the only countries to have their own distinct group (Sproesser et al., 2022). Interestingly, clusters were more different in relation to food traditions than modern ways, showing how modern foods and culture are being standardized. The top food traditions were “Eating food that has been prepared in grandmother’s way” and “Only women do the cooking”, whereas the top modern ways were “Eating fast food (e.g., hamburgers)” and “Eating food from vending machines (e.g., chips)”. In Cameroon, medical students exhibited a high prevalence of disordered eating habits, including meal skipping, irregular meals, low fruit and vegetable consumption, and a high intake of candy, fried foods and alcohol (Bede et al., 2020). A recent study in Spain found that dietary pattern changes and binge eating may be major contributing factors to eating disorders in university students (Eguren-Garcia et al., 2024). Medical students and university students may not prioritize diet quality and eat only when their schedule allows. Food behaviors may be important additions to future studies.

### 4.9. Impact of Social Media

Traditional screening tools may not capture the positive or negative effects of social media use. Social media has become ingrained in contemporary society (Wani et al., 2024). There is a major concern regarding its propensity to spread health misinformation (Denniss & Lindberg, 2025; Trethewey, 2019). Furthermore, misinformation seems to be focused on physical activity and fitness, dietary behaviors and nutrition, psychological health and well-being, and substance use and risky behavior (Paul & Headley-Johnson, 2025), which are all related, directly or indirectly, to eating disorder risk. The prevalence of body dysmorphic disorder was 6.7% in female Egyptian medical students who use social media (Abdelaziz et al., 2025). A prevalence of 4.65% was found among medical students in Pakistan (Hameed et al., 2025); however, it was much higher (46.1%) among female Indian medical students who also reported high social media use (Sekhar et al., 2025). Since the onset of the COVID-19 pandemic, social media consumption by female medical students has significantly increased (Nisar et al., 2022), with 95.9% of medical students self-reporting frequent consumption of social media (Pronk et al., 2021). A survey of Iranian medical students found that social media was used as a stress-coping mechanism (Shiraly et al., 2024). Furthermore, in Jordanian medical students, increased social media use was negatively associated with academic performance due to its distracting nature (Taha et al., 2025). A similar result was found in Iranian medical students; however, males were more at risk (Azizi et al., 2019). Problematic social media use, depression and anxiety were associated with a higher risk of BN in Lebanese medical students (Sfeir et al., 2023). Interestingly, studies on social media use appear to only focus on associations with negative psychological health (Dibb, 2019) rather than physical health outcomes. Although the pressures from social media play a crucial role in female medical students’ perceptions of themselves (Pop et al., 2021), there is a lack of evidence suggesting that it significantly contributes to the higher risk of eating disorders. Regardless, social media use is consistently associated with negative body image, and longitudinal studies suggest that this association will strengthen over time (Fardouly & Vartanian, 2016). While social media use has not resulted in an overall negative effect for medical students, to date, it does negatively affect study and sleep habits (Neel Rajendrakumar Barot et al., 2025). Medical students will have to be competent in the use of social media as it will inevitably become more important in the future of healthcare (Jain et al., 2024). However, future studies will need to include social media use as a variable so it can be monitored.

### 4.10. Expand Screening to All Eating Disorders

There are limitations in the types of eating disorders that are prevalent in medical students. While screening tools attempt to identify behaviors related to a range of eating disorders, it may benefit the field to elucidate the types of eating disorders that medical students are most at risk of developing, considering BED and OSFEDs are the most common (Santomauro et al., 2021). Medical students may also be at a higher risk of night eating syndrome (NES), given their erratic schedules. In central India, 13.8% of medical students were at risk of NES, with 10.1% being considered mild (Singh et al., 2025), with insomnia being the main cause. Similarly, 8.8% of Turkish medical students were diagnosed with probable NES, which was associated with being a morning type, poorer sleep quality, impulsive, depressed and anxious (Yucel et al., 2025). However, assessing sleep quality may also be important in medical students. Poor sleep quality was reported by 62.2% of Latin American medical students. While a high level of resilience was associated with better sleep quality, being female and depressive and anxious symptoms were associated with poor sleep quality (Valladares-Garrido et al., 2025). This shows that more eating disorder types need to be included in future research.

## 5. Discussion

A narrative review was chosen as there were several broad questions to answer; systematic reviews focus on a very specific question. In addition, the goal was to find new insights into the issue of elevated eating disorder risk in medical students, a task that is best suited to narrative reviews (Sukhera, 2022). This review briefly discussed the risks identified by the included studies on eating disorder risk in medical students; a full-depth analysis of all potential factors was beyond the scope of this manuscript.

**Figure 1:**
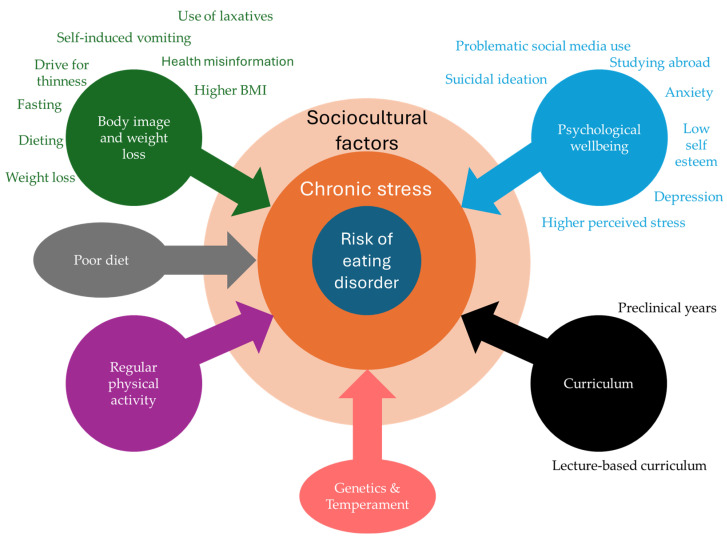
The risk factors contributing to eating disorder risk in medical students worldwide.

The risk of eating disorders is higher in medical students compared to the general population. It is also higher in university students (Daly & Costigan, 2022). While this review highlights four key factors to disordered eating behaviors among medical students, including body image and weight loss, regular physical activity, curriculum and psychological well-being, it is essential to note that no single risk factor universally accounts for the higher rate of eating disorder risk in medical students worldwide. More importantly, these risk factors are tightly interconnected. Overall, the diagnosis of an eating disorder is greater than the sum of its parts. This point is reinforced by a meta-analysis that demonstrated that no convincing evidence pointed to a single risk factor for an eating disorder (Solmi et al., 2021). Furthermore, the highly individualized nature of eating disorders suggests risk factors are personal. However, chronic stress, usually referred to as stress, may be the most important contributor due to its physiological and psychological health effects. Nevertheless, it may be important to begin to focus on chronic stress. Thus, while stress in medical training environments may be a concerning contributor to the development of eating disorders among medical students, it must be viewed within the broader context of the nuanced landscape of eating disorder risk. We summarized the potential risk factors in Figure 1. Overall, these data support the premise that medical school may result in a perfect storm and create the ideal environment for eating disorder risk. The high levels of chronic stress and professional expectations in medical students might represent stronger risk factors compared to other populations.

This review is a call to action, highlighting the goal to prioritize student wellness in medical school. Despite the evidence that eating disorder risk is higher in medical school and that medical students receive education on eating disorder diagnoses and treatments, medical students are not aware of their personal eating disorder risk. Awareness is also a concern for practicing physicians. While emergency departments may not prioritize knowledge of eating disorders, 95% of emergency physicians were not aware of the published practice guideline for treating eating disorders in emergency departments, while less than 2% received a residency rotation on eating disorders (Ma et al., 2021). Moreover, the rising number of female physicians seeking treatment for eating disorders suggests that many of these struggles may originate during medical school and persist silently throughout training (Rimmer, 2014). While the data on direct career impact is limited, the stigma surrounding mental health, particularly among high-achieving professionals, may result in delayed help-seeking, increased burnout, and reduced professional longevity.

In the future, it may be commonplace for some medical students to require treatment for eating disorders during their studies or clinical training (Dent, 2012). Before this happens, systemic changes within universities are required. All university students should receive information and training on eating disorders. This will help individuals to recognize signs in themselves and their classmates. Medical students and students from other healthcare fields and high-intensity degrees should have more in-depth eating disorder training to further increase awareness. Student and faculty peer leaders and mentors will need training to recognize warning signs and guide students to resources. Confidential screening for eating disorders should be available and integrated into wellness checks. Student health information needs to be firewalled from academic records to reduce fear of repercussions. This may help increase early detection for better treatment outcomes. Clinicians with expertise in eating disorders should be available to help diagnose and treat at-risk students, especially with flexible appointment options, including telehealth and after-hours services, with guaranteed confidentiality protections. Medical schools and other healthcare-related fields should integrate eating disorders, body image, nutrition myths, and compulsive exercise into preclinical curricula. In addition, content on stress management, including physical activity, self-compassion, and cognitive perfectionism, can be added to the curriculum. Furthermore, weight stigma and biased health messaging, especially in anatomy, nutrition, and population health courses, will need to be included in curricula. High-pressure, appearance and performance-focused cultures are the perfect environment for eating disorders. Promoting a learning environment that emphasizes health and well-being, including protected meal breaks, access to food during long academic or clinical days, and reasonable scheduling, as well as time off for treatment, will help prevent eating disorders and may also reduce burnout. Medical school curriculum teams may also consider incorporating more problem-based learning into the preclinical years and providing medical students with more opportunities for professional identity development.

## 6. Conclusions

In conclusion, eating disorder risk among medical students warrants greater attention from educators, student health systems, and researchers. This review supports a multifactorial model in which chronic stress is an important overarching target, where perfectionism, body image, physical activity, psychiatric comorbidity, and sociocultural influences are contributors. Future work should move beyond prevalence estimates alone toward longitudinal designs, better sex- and gender-inclusive reporting, and intervention studies that identify which modifiable factors can be targeted most effectively during medical training. In particular, developing integrative strategies that address chronic academic stress, body image pressures, mental health, and institutional culture may help clarify how medical schools can better support student well-being and reduce risk over time; ultimately producing physicians better equipped for contemporary healthcare.

## Figures and Tables

**Table 1:** Eating disorder prevalence and statistically significant risk factors in medical students in alphabetical order by country.

Country	Gender ^†^	Sample Size ^†^ (n)	Age * (Years)	Prevalence	Instrument Used	Risk Factors for Eating Disorders
Brazil (Alberton et al., 2013)	♂ and ♀	391	≥18	9.97%	EAT-26	Female, high BMI, preclinical years, aged 20 years or less
	♂	191		2.61%		
	♀	200		17.00%		
Brazil (Bosi et al., 2016)	♀	202	21.8 ± 2.8	9.90%	EAT-26	Body image dissatisfaction, inadequate eating practices
Chile (Mandiola et al., 2022)	♀	163	18–24	23.90%	EAT-26	Academic stress, perfectionism, social anxiety
Egypt (Ali & Shehata, 2020)	♂ and ♀	615	≥18	33.00%	EAT-26	Female, preclinical years, not married, high BMI, lower physical activity among at risk
	♂	202		28.70%		
	♀	413		35.10%		
Egypt (Elsherif et al., 2025)	♂ and ♀	370	22–25	36.76%	EAT-26	Female, high BMI, first-born, smoking, orlistat use, alexithymia, social cognitive impairment
	♂	136		14.70%		
	♀	234		85.30%		
France (Tavolacci et al., 2025)	♂ and ♀	7506	≥18	26.20%	SCOFF	Female, non-binary, preclinical years, financial difficulty, humiliation, sexual violence, sexual assault, depression, anxiety, suicidal thoughts, alcohol/tobacco use, foregoing healthcare for financial or academic reasons
	♂	2132		14.40%		
	♀	5321		30.90%		
	NB	53		26.00%		
India (Perti et al., 2026)	♂ and ♀	319	23.5 ± 1.56	26.33%	EAT-26, SCOFF	High BMI, body shape, psychological distress
	♂	159		26.41%		
	♀	160		26.25%		
India (Mallaram et al., 2023)	♀	777	20.62 ± 1.54	7.50%	EAT-26	High BMI, body image concerns, low self-esteem
Iran (Naeimi et al., 2016)	♂ and ♀	430	21.09 ± 2.24	9.50%	EAT-26	Low self-esteem, body image, mothers’ education level
	♂	134		7.50%		
	♀	296		10.50%		
Kingdom of Bahrain (Almahmeed et al., 2025)	♂ and ♀	397	≥17	32.10%	EAT-26	Preclinical years, living alone or with family, previously sought help for mental health, COVID-19 pandemic
Kingdom of Saudi Arabia (Ghamri et al., 2022)	♂ and ♀	417	21.65 ± 1.51	32.10%	EAT-26	Female, preclinical years, high BMI
	♂	138		25.36%		
	♀	279		35.48%		
Kingdom of Saudi Arabia (Aljaffer et al., 2026)	♂ and ♀	425	21.23 ± 1.50	49.60%	EAT-26	High BMI, anxiety, depression
	♂	98		46.40%		
	♀	113		53.60%		
Lebanon (Bizri et al., 2020)	♂ and ♀	131	≥21	17.00%	EAT-26	Female, aged 22–25 years, recent stressors, currently seeking mental health provider, history of mental health diagnosis, family history of eating disorder
	♂	61		27.30%		
	♀	70		72.70%		
Lebanon (Sfeir et al., 2023)	♂ and ♀	363	22.65 ± 3.48	33.60%	EAT-26, EAT-26 bulimia subscale	Problematic social media use, body dissatisfaction, depression and anxiety associated with higher risk of bulimia
Malaysia (Ngan et al., 2017)	♂ and ♀	263	20–28	11.00%	EAT-26	High BMI, unsatisfactory social relationship with friends
	♂	92		10.80%		
	♀	171		11.10%		
Morocco (Azzouzi et al., 2019)	♂ and ♀	710	21.27 ± 2.02	32.80%	SCOFF	Female, weight control behavior, dieting, fasting-induced vomiting, appetite suppressants, EDI-2
	♂	248		23.70%		
	♀	462		37.60%		
Norway (Rostad et al., 2021)	♂ and ♀	1044	24.9 ± 3.1	18.30%	EDS-4	Female, high BMI, curriculum type, medical school stress, metal distress
	♂	304		5.60%		
	♀	740		23.50%		
Pakistan (Memon et al., 2012)	♂ and ♀	435	18–25	22.75%	EAT-26	Female, high BMI
	♂	93		12.90%		
	♀	342		25.40%		
Peru (Carpio-Oviedo et al., 2025)	♂ and ♀	190	Med 23	34.72%	EAT-26	Moderate to severe academic stress, having breakfast more than half the time in a week
	♂	76		35.50%		
	♀	114		35.10%		
Uganda (Abaatyo et al., 2025)	♂ and ♀	224	27 ± 5.6	16.50%	SCOFF	Female, >1 current sexual partners, suicidal ideations
	♂	141		14.20%		
	♀	83		20.50%		
United States (Hammad et al., 2025)	♂, ♀ and NB	376	≥18	24.30%	SCOFF	Weight bias internalization
United States (Cummings & Uhley, 2024)	♀	69	N/A	14.50%	EAT-26	Not receiving mental health treatment
Meta-regression—6 countries (Jahrami et al., 2019a)	♂ and ♀	3520	19–23	10.50%	EAT-26	Age × gender × high BMI
Meta-analysis—8 countries (Jahrami et al., 2019b)	♂ and ♀	5722	18.5–25	10.40%	EAT-26, EDI-1, SCOFF	Female
	♀	1849		13.70%		
Meta-analysis—20 countries (Fekih-Romdhane et al., 2022)	♂ and ♀	21,383	Med 21.8	17.35%	EAT-26, SCOFF, EDI-1/2, EDE-Q, ANIS and ORTO-15	High BMI, non-Western culture, geography
Cross-sectional study—12 countries (Belhaj Salem et al., 2025)	♂ and ♀	5061	18–36	24.80%	EAT-26	Female, lack of regular sport activity, parents pushed to eat, food insecurity, sleep disorder, underweight, daily exposure to thin ideal, past suicide attempts, not satisfied with weight, borderline personality disorder, schizophrenia, IBD, T1DM
	♂	1765		22.00%		
	♀	3296		26.20%		

^†^ Gender and sample size are reported as presented in the original paper or from data in the original tables. For studies with male and female participants, separate data are presented if available, as well as the total for males and females. * Ages as reported in the original manuscript, either as a range, mean, mean ± SD, median (Med), or best estimate determined from the details in the paper. ANIS = Anorexia Nervosa Inventory for Self-Rating; EAT-26 = Eating Attitudes Test-26 Item; EDI-1 = Eating Disorder Inventory 1st edition; EDI-2 = Eating Disorder Inventory 2nd edition; EDS-4 = Eating Disturbance Scale with 4 subscales; EDE-Q = Eating Disorder Examination-Questionnaire; IBD = Inflammatory Bowel Disease; N/A = not available; NB = non-binary; ORTO-15 = 15 item questionnaire for the diagnosis of orthorexia; SCOFF = Sick, Control, One, Fat, Food Questionnaire; T1DM = type 1 diabetes mellitus.

## Data Availability

No new data were created or analyzed in this study. Data sharing is not applicable to this article.

## Abbreviations

The following abbreviations are used in this manuscript:

AN	anorexia nervosa
ANIS	Anorexia Nervosa Inventory for Self-Rating
BED	binge eating disorder
BITE	Bulimic Investigatory Test, Edinburgh
BN	bulimia nervosa
DSM-5	Diagnostic and Statistical Manual of Mental Disorders
EAT-26	Eating Attitudes Test—26 Item
EDI-1	Eating Disorder Inventory First Edition
EDI-2	Eating Disorder Inventory Second Edition
EDS-4	Eating Disturbance Scale with four subscales
EDE-Q	Eating Disorder Examination-Questionnaire
NB	non-binary
ORTO-15	15 item questionnaire for the diagnosis of orthorexia
OSFED	other specified feeding or eating disorder
SCOFF	Sick, Control, One, Fat, Food Questionnaire
